# Protection Function and Mechanism of Rosemary (*Rosmarinus officinalis* L.) Extract on the Thermal Oxidative Stability of Vegetable Oils

**DOI:** 10.3390/foods12112177

**Published:** 2023-05-28

**Authors:** Xiaoxiao Song, Xiaonan Sui, Lianzhou Jiang

**Affiliations:** 1 State Key Laboratory of Food Science and Resources, China-Canada Joint Laboratory of Food Science and Technology (Nanchang), Key Laboratory of Bioactive Polysaccharides of Jiangxi Province, Nanchang University, Nanchang 330047, China; songxiaoxiao@ncu.edu.cn; 2 College of Food Science, National Research Center of Soybean Engineering and Technology, Northeast Agricultural University, Harbin 150030, China

**Keywords:** rosemary extract, vegetable oil, physicochemical indexes, induction period, kinetic parameters

## Abstract

Rosemary (*Rosmarinus officinalis* L.) extract (RE) is one of the most efficient natural antioxidants and can significantly inhibit oil oxidation during storage or heating. The present study determined the protective capacity and mechanism of RE on the thermal oxidative stability of different vegetable oils by adding RE (70% carnosic acid) to five types of vegetable oils (soybean oil, rapeseed oil, cottonseed oil, rice bran oil, and camellia oil) and measuring the physicochemical indices (fatty acid composition, tocopherol content, total phenolic content, and free radical scavenging capacity), induction period, and thermal oxidative kinetic parameters. The relationships between the antioxidant capacity and thermal stability parameters were determined. The results show that, compared with artificial antioxidants, RE significantly increased the free radical scavenging capacity, induction period, and activation energy (*Ea*) of thermal oxidation, decreasing the thermal oxidation reaction rate (k) of all vegetable oils, especially rice bran oil. A Spearman correlation analysis showed that the induction period (IP) and *Ea* showed a significant positive correlation, the combination of which effectively reflected the efficiency of antioxidants and explained the inhibition mechanism of RE towards oil thermal oxidation.

## 1. Introduction

Vegetable oils and fat-based foods easily oxidize and deteriorate during processing, storage, and cooking when exposed to an oxidizing atmosphere. This diminishes the nutritional value of lipids, causing economic losses [1] and the production of toxic substances that pose a threat to human health [2]. Artificial antioxidants are frequently used in the food industry. However, excessive use of some low-price artificial antioxidants (e.g., butylated hydroxyanisole [BHA] and butylated hydroxytoluene [BHT]) can lead to chronic diseases [3] and potentially provoke the onset of degenerative diseases [4]; thus, they are restricted by many countries. A widely adopted solution is to add antioxidants from natural sources, such as rosemary extracts [5,6], phenolic extract of camellia oleifera seed cake, and tea polyphenol palmitate, among others [7], to both improve the antioxidant capacity and enhance nutritional value.

Rosemary (*Rosmarinus officinais* L.) extract (RE) is a natural antioxidant that has been recognized by worldwide food regulations as a generally recognized as safe (GRAS) additive [8] because of its relatively strong antioxidant capacity and fat solubility. It is regarded as a natural alternative to artificial antioxidants to minimize oxidative reactions and extend shelf life while offering a high degree of consumer acceptance [9]. Previous research has found that rosemary exhibits higher antioxidant activity in moringa oil at 60 °C compared with sage and savory summer extracts [10]. Similar results have been reported by other researchers [11,12,13]. In addition, soybean oil, cottonseed oil, and rice bran oil with RE exhibited improved characteristics (i.e., higher induction period, antioxidant capacity, total phenolic content, and longer shelf life) compared with those treated using artificial antioxidant complexes (BHA and BHT) during accelerated storage. RE effectively inhibits the formation of lipid hydroperoxides [5]. Other studies have shown that RE can improve the oxidative stability of hemp seed oil [14] and microencapsulated fish oil [15] by inhibiting the chain reaction of lipid oxidation. However, the mechanism of the lipid oxidation inhibition capacity of RE still needs to be further analyzed by means of thermal oxidation kinetics measurements because most lipid processing and application processes require heating, which could accelerate the oxidation reaction. However, the existing research is insufficient.

Rancimat and differential scanning calorimetry (DSC) are widely used to determine the oxidative stability of edible oils. Compared with the traditional Schaal oven and forced-air oven tests, these approaches do not involve toxic chemicals, making their use more efficient with less pollution or waste [16,17,18]. Rancimat could be listed as the Chinese national standard for determining the oxidative stability of fats or oils. DSC is a widely used thermal analysis technique for lipids; this is because lipid oxidation is an exothermic reaction, so it provides heat that can be measured using a DSC thermal analyzer [19]. Meanwhile, when using the non-isothermal method of adjusting different heating rates, exothermic peaks (initial oxidation temperature and initial decomposition temperature) of different reaction stages of lipid oxidation have been observed [20,21]. When comparing the DSC and Rancimat methods for evaluating the oxidative stability of three types of oil, it was demonstrated that the DSC method is more efficient in terms of time and sample consumption [22]. The effect of adding RE to edible oils under pressurized DSC and Schaal oven conditions has also been demonstrated [4]. However, there are differences among the different vegetable oils due to their endogenous components. To the best of our knowledge, previous studies have not conducted a comprehensive comparative evaluation of RE protection and have not directly demonstrated the oxidation kinetic parameters such as *Ea* through a co-analysis of the Rancimat and DSC methods. Whether the parameters of different indices are correlated needs to be further clarified.

Therefore, in this study, we tested five types of vegetable oil (soybean, rapeseed, rice bran, cottonseed, and camelia oil) that are widely used in China. RE and two artificial antioxidants (BHA and BHT) were added to the oils, and their physicochemical indices (fatty acid composition, total phenol content, tocopherol content, and monomer content) were determined. Rancimat and non-isothermal DSC methods were adopted to explore the oxidative induction period (IP) and calculate the thermal oxidation kinetic parameters, respectively. Finally, a Spearman correlation analysis was used to analyze the correlations between thermal oxidation kinetic indices and lipid physicochemical indices. This study provides a theoretical basis for the further development and application of RE as a lipid antioxidant.

## 2. Materials and Methods

### 2.1. Materials

Soybean oil, rapeseed oil, cottonseed oil, rice bran oil, and camellia oil without additional antioxidants were provided by Hao Koufu Co., Ltd. (Yichun, China). Commercial RE with a very high carnosic acid content (70%) was purchased from Hainan Shupu Science and Technology Co., Ltd. (Haikou, China). BHA, BHT, DPPH, and ABTS were purchased from Sigma-Aldrich (St. Louis, MO, USA). All of the other chemicals and reagents were of analytical grade.

RE was directly added to the oils at a concentration of 400 mg/kg (the added content was below the regulated limit set by the China National Standard GB 2760-2014 (700 mg/kg)), and the mixture was stirred for 10 min at room temperature. As a positive control, a mixture of synthetic antioxidants (*w*/*w* = 1:1) was added at a legal limit of 200 mg/kg [23] as a positive control, which met the maximum permitted amount of the China National Standard (GB 2760-2014). Antioxidant-free oils were used as blank controls.

### 2.2. Initial Indexes Analysis

#### 2.2.1. Fatty Acid Composition Analysis

The fatty acid composition was analyzed using an Agilent 7890 gas chromatograph coupled with an Agilent 5975 mass spectrometer (GC-MS; Agilent Technology, Santa Clara, CA, USA) equipped with an HP-88 capillary column (100 × 0.25 mm). The fatty acid composition was analyzed as described in our previous study [5,24]. In brief, to prepare fatty acid methylesters, oils were saponified and then methylated in methanol. At injection temperature 250 °C, the carrier gas was helium (100 kPa) and the split ratio was 1:30. The oven temperature was programmed as described in our previous work [14].

#### 2.2.2. Measurement of Tocopherol Isomers

Tocopherol content was measured according to a previously described method [25] and which had been detailed in our previous work [14]. Finally, Tocopherols were quantified using a fluorescence detector at excitation and emission wavelengths of 290 and 325 nm, respectively.

#### 2.2.3. Total Phenolic Content Analysis

The total phenolic content of the oils was measured using the Folin–Ciocalteu method according to a previous work [26] with modifications [14]. The total phenolic content of the oil samples were expressed as gallic acid equivalents (mg/mL).

#### 2.2.4. Determination of DPPH and ABTS Antioxidant Capacity

The DPPH antioxidant and ABTS assays were performed as previously described [14,27,28]. Briefly, a spectrophotometer (UV Mini 1240, Shimadzu, Kyoto, Japan) was adopted. The total antioxidant capacity obtained using the ABTS assay was reported as milligrams of Trolox equivalents per milliliter of sample.

### 2.3. Rancimat Analysis

The Rancimat method was performed under isothermal conditions at 120 °C in an oxygen atmosphere. The Rancimat analysis was conducted using a Rancimat 892 instrument (Metrohm, Herisau, Switzerland). Oils (5.0 g each) were accurately weighed in each reaction vessel. The target temperature was set at 120 °C and the airflow rate was 20 L/h. The results are expressed as the IP [29], which was automatically determined from the inflection point of the curve using software supplied by the company. To assess the protective effects of RE, the protection factor (PF) can be calculated from the IP [30]:

(1) $$\mathrm{PF}=\frac{\mathrm{IP}\left(\mathrm{oil}\;\mathrm{sample}\right)}{\mathrm{IP}\left(\mathrm{control}\right)\;}$$

Similarly, the percentage antioxidant activity (%AA) was determined according to the method of Ramamoorthy and Bono [31] as follows:

(2) $$\%\mathrm{AA}=\frac{\mathrm{IP}\left(\mathrm{oil}\;\mathrm{sample}\right)-\mathrm{IP}\left(\mathrm{control}\right)}{\mathrm{IP}\left(\mathrm{positive}\;\mathrm{control}\right)-\mathrm{IP}\left(\mathrm{control}\right)}$$

### 2.4. DSC Analysis

DSC was used to calculate the thermal stability of each oil sample, and 5 mg of the oil sample was added to an aluminum crucible. All of the thermal experiments were conducted using a NETZSCH STA 449 F3 instrument (Selb, Germany) by increasing the temperature from 30 to 400 °C at an oxygen flow of 100 mL/min and heating rate of 3, 6, 9, 12, and 15 °C. According to a non-isothermal test, the kinetic equation for thermal oxidation [32] is:

(3) $$\frac{\mathrm{di}}{\mathrm{dT}}=\frac{A}{\beta }\exp\left(-\frac{Ea}{\mathrm{RT}}\right)f\left(i\right)$$

where i is the extent of the reaction, which can be determined from DSC and present as signal changes; A is the pre-experimental factor (s^−1^); T is the absolute temperature (K); β is the heating rate; *Ea* is the apparent activation energy (kJ/mol); R is the gas constant (8.314 J/mol·K); and f(i) describes the dependence of reaction rate on the extent of the reaction.

In this study, a characteristic point was observed in the DSC curves during oil oxidation: the onset temperature (i.e., initial oxidation temperature) [33]. In our study, the Ti of the DSC curves was selected to calculate the kinetic parameters by adopting the OFW model-free equation, which is described as follows:

(4) $$\mathrm{Log}\beta =\log\left(\frac{AEa}{\mathrm{Rg}\left(i\right)}\right)-2.315-0.4567\frac{Ea}{\mathrm{RT}}$$

*Ea* can be determined from the slope of the curve by plotting logβ against 1/T under a given value of i.

### 2.5. Statistical Analysis

All sampling and chemical analyses were performed in triplicate, and all results are expressed as the mean of triplicate values with standard deviations. Statistical analyses were conducted on the obtained results using a one-way analysis of variance (ANOVA), Duncan’s multiple range test, and Spearman’s correlation analysis using SPSS V. 22 software (IBM Corporation, New York, NY, USA).

## 3. Results and Discussion

### 3.1. Physicochemical Indices

#### 3.1.1. Fatty Acid Composition

Table 1 exhibits the fatty acid compositions of the five vegetable oils. Cottonseed oil contained more saturated fatty acid (SFA) than the other oils, while camellia oil and rape seed oil contained more monounsaturated fatty acid (MUFA). Soybean and cottonseed oil had more polyunsaturated fatty acid, which is essential for skin and hair health and cardiovascular and cerebrovascular diseases [21]. Although oils containing high amounts of linoleic and linoleinic acid are prevalent in daily life, the saturation degree of fatty acids not only determines the nutritional value of vegetable oils but can also affect their oxidative stability; the higher the degree of unsaturation, the more easily that fatty acids can be oxidized [34,35].

#### 3.1.2. Tocopherol Isomer Content

Tocopherol, a common fat-soluble vitamin in vegetable oil, not only has important biological functions but also plays an important role in inhibiting lipid oxidation. The tocopherol isomer contents of the five vegetable oils are shown in Table 1. The cottonseed, soybean, and rapeseed oils had the highest total tocopherols, most of which were (β + γ)-tocopherol. The cottonseed, rice bran, and camellia oils had the highest α-tocopherol.

### 3.2. Antioxidant Capacities and Total Phenolic Content

**Figure 1:**
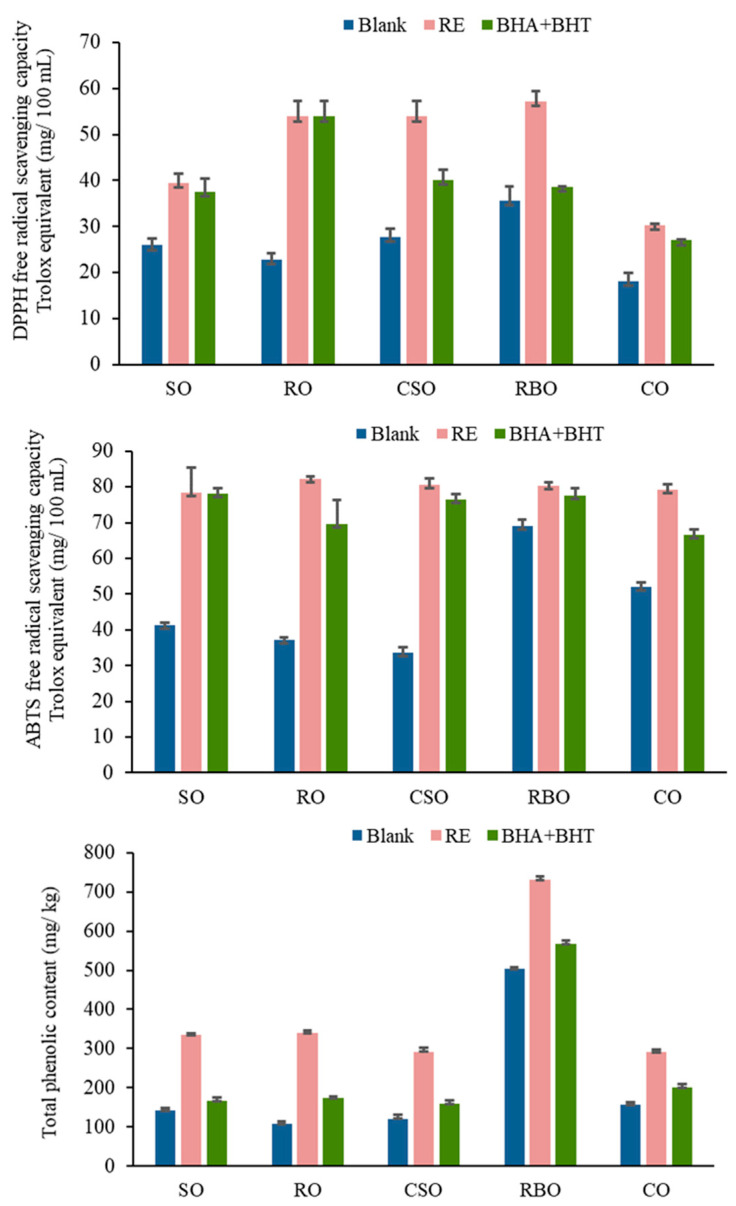
Comparation of antioxidant capacity and total phenolic content (TPC) of vegetable oils without rosemary (*Rosmarinus officinalis* L.) extract (RE) or butylated hydroxyanisole (BHA) + butylated hydroxytoluene (BHT), with RE, and with BHA + BHT.

From the antioxidant capacity indices (Figure 1), the DPPH and ABTS scavenging abilities were not consistent, which may be due to the different inhibitory mechanisms of each free radical. After the addition of RE, the total phenolic content and free radical scavenging ability significantly improved. Interestingly, rice bran oil had the highest total phenolic content, lowest total tocopherol content, and strongest DPPH and ABTS radical scavenging abilities among the five vegetable oils. This might reflect the presence of gamma oryzanol in rice bran oil, which reportedly has a strong antioxidant capacity and can effectively inhibit lipid oxidation [5]. Cottonseed oil had a poor radical scavenging capacity despite possessing the highest total tocopherol content among the five vegetable oils. Camellia oil had a relatively strong ABTS radical scavenging ability but a poor DPPH radical scavenging ability. These results indicate that vegetable oils are complex systems and that the reaction path is not determined by only a single antioxidant component [36,37].

### 3.3. Rancimat Analysis

Rancimat is widely used to measure the oxidative stability of oil samples and the efficiency of antioxidants. The time that elapses until these secondary reaction products appear, the IP [29], is an important parameter for the oxidative assessment of animal fats and vegetable oils [38]. The higher the IP of the oil sample with added antioxidants compared with the control, the better the antioxidant activity [38]. Significant changes (*p* < 0.05) were observed in the oxidative stability of the blank oil samples after the addition of RE and synthetic antioxidants (BHA + BHT), as shown in Table 2. As expected, oils without antioxidants were much easier to oxidize under harsh conditions; hence, the lowest IP values were obtained. In addition, the PF calculated via the IP revealed that RE could act as an effective protector against the oxidation of oil samples compared with synthetic antioxidants [39]. Unlike the PF, the %AA revealed differences in the effects of adding RE to the five oil types. Notably, the RBO generated the most and initiated the strongest synergistic effects among the oils (6.50), and the CO had the weakest synergistic effect (2.94).

The DPPH and ABTS scavenging capacities of the RBO groups did not significantly increase compared with the %AA results, but this was not contradictory. There are different antioxidant evaluation systems. The former is the scavenging capacity of the sample for different free radicals in different reaction systems, whereas the latter is the ability of the sample to inhibit the thermal oxidation of lipids.

### 3.4. Non-Isothermal DSC Analysis

#### 3.4.1. Initial Oxidation Temperature

**Figure 2:**
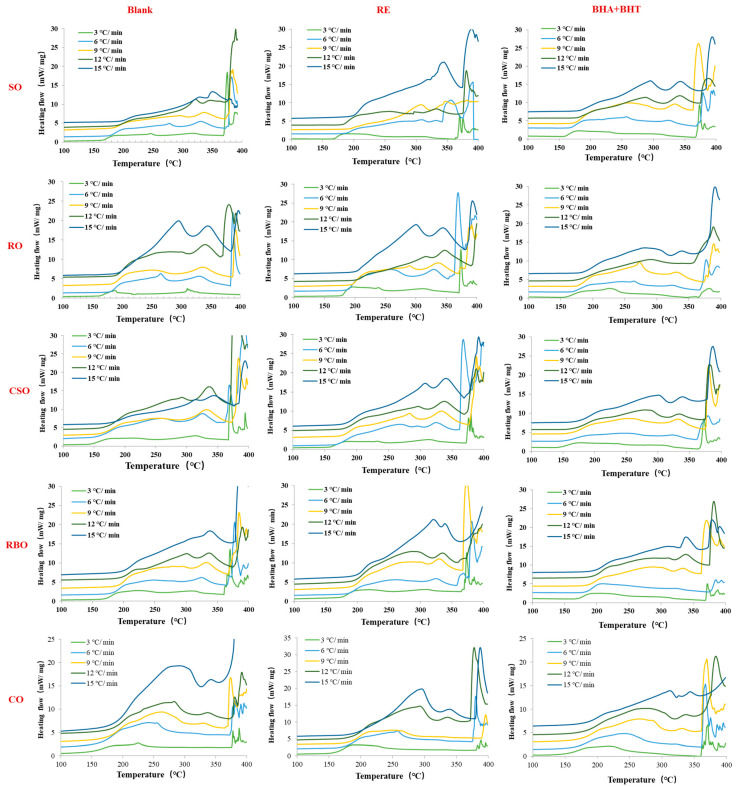
Differential scanning calorimetry (DSC) thermograms of oil samples under different heating rates.

A non-isothermal DSC analysis was conducted to determine thermal oxidative stabilities and oxidation kinetics. As shown in Figure 2, an apparent exothermic curve was observed for all samples in an oxygen atmosphere. From the resulting curve, the first point of the thermal response, namely the intersection of the extrapolated baseline and the straight tangent (leading edge) of the exothermic peak, is the initial oxidation temperature (IOT).

The IOTs of the oil samples increased with an increase in the heating rate (Table 3) [40]. At a low heating rate, hydrogen peroxide rapidly reacts with excess oxygen to form low-molecular weight compounds in oil systems, accelerating the degradation process [21]. Under a high heating rate, intermediate products can easily volatilize directly rather than proceeding further in the oil [40].

On this basis, we used the IOT to conduct further analyses. The addition of antioxidants did not increase the IOT of the vegetable oil samples. RE only affected soybean oil, rapeseed oil, and cottonseed oil (*p* < 0.05), whereas synthetic antioxidants only affected soybean oil (*p* < 0.05). Rice bran oil exhibited the highest IOT among the vegetable oils, which is in accordance with the IP results.

#### 3.4.2. Activation Energy, Pre-Exponential Factor, and Reaction Rate Constant

Table 4 shows the kinetic parameters (k at 180 °C) calculated from the IOT of different samples under different heating rates; the corresponding scatter fitting curves are shown in Figure S1. The correlation coefficients were calculated using the Ozawa–Flynn–Wall method and Arrhenius equation for the *Ea*, pre-exponential factor (A), and rate constant (k) at 180 °C (a commonly used frying temperature; at frying temperatures of >200 °C, acrylamide forms). Among all the blank oil samples, rice bran oil exhibited the highest *Ea* value (88.69 kJ/mol) and the lowest k value (0.25). Simultaneously, the stability scale for the five oils without any antioxidant was RBO > SO > RO > CSO > CO, indicating that RBO is the most stable among the tested oil types during thermal oxidation. As expected, oil samples with incorporated RE or synthetic antioxidants (BHA + BHT) showed higher *Ea* values than the blank. Moreover, despite the presence of CO, all oil samples with RE exhibited higher *Ea* values than the synthetic antioxidants incorporated. The pre-exponential factor of the oil samples also significantly changed compared with the oil samples with RE or synthetic antioxidants incorporated. However, the pre-exponential factor is only affected by the chemical reaction itself and changes accordingly when the reaction system is altered. In other words, these results may be due to the decrease in activated complex formation in the presence of the antioxidant, which combines (a) the reduction of free radicals by donating hydrogen and (b) the rotational freedom in the transient activated complexes [33]. The addition of RE also decreased the k value, which slowed the process of lipid thermal oxidation. As expected, the results also showed that oil with RE was more effective than the incorporated synthetic antioxidant, which might reflect the influence of synthetic antioxidants. BHA and BHT are relatively susceptible to oxidation and volatilization [41]. Under harsh heating conditions, the effective element of the antioxidant escaped from the oil sample and was oxidized. Thus, the phenolic compounds either decomposed or reacted with other substances in the test medium, further confirming the thermal stability of RE.

### 3.5. Correlation Analysis

**Figure 3:**
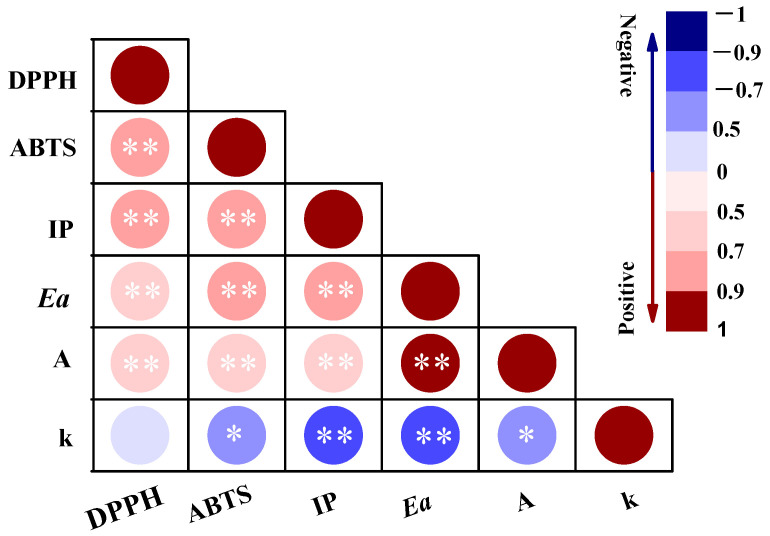
Spearman correlation analysis for the relationships among the free radical scavenging capacities, induction period (IP), and oxidative kinetic parameters of different oil samples. * representing for *p* < 0.05, ** representing for *p* < 0.01.

To further determine the relationship among the free radical scavenging capacities, IP, and oxidative kinetic parameters of the oil samples, a Spearman correlation analysis was conducted (Figure 3). DPPH exhibited significant positive correlations with the ABTS free radical scavenging capacities, the IP, and the *Ea*, with correlation coefficients (ρ) of 0.781 (*p* = 0.001), 0.735 (*p* = 0.001), and 0.699 (*p* = 0.004), respectively. Similar results were observed for the ABTS free radical scavenging capacity. This indicates that the antioxidant capacity correlates well with the thermal oxidation stability of these oils, possibly because antioxidant compounds can effectively block the occurrence of thermal oxidation reactions and increase the oxidation IP and *Ea* of the reaction. Furthermore, the IP was also significantly positively correlated with the *Ea* (ρ = 0.793, *p* = 0.0002) and negatively correlated with k (ρ = −0.719, *p* = 0.003). This is in accordance with other studies that have reported that the Rancimat test and DSC technique are complementary techniques during oil oxidation assessments [42]. However, the strong positive correlation between the *Ea* and A may have occurred because A was calculated from the *Ea* using a relatively simple equation.

## 4. Conclusions

In this study, the protective effects and mechanisms of RE on the thermal oxidation stability of five common commercially available vegetable oils were analyzed. We found that RE effectively improved the thermal oxidation stability and antioxidant activity of all five vegetable oils by increasing the thermal oxidation *Ea* and by reducing the reaction rate. A correlation analysis demonstrated that under such a system, the free radical scavenging ability, oxidation IP, thermal oxidation stability, and other indicators showed a significant positive correlation. However, further investigation is required to determine whether the endogenous antioxidant components present in the oil have an impact on the protective efficacy of RE. Additionally, it is necessary to explore the potential synergistic or antagonistic effects between these components and RE under various antioxidant evaluation systems, as well as their underlying mechanisms.

## Figures and Tables

**Table 1:** Fatty acid composition and tocopherol isomer content of five vegetable oils.

Indices	SO	RO	CSO	RBO	CO
*Fatty acid composition (g/100 g)*					
Myristic acid	0.08 ± 0.01	0.07 ± 0.01	0.67 ± 0.06	0.26 ± 0.01	nd
Palmitic acid	9.12 ± 0.15	4.84 ± 0.10	22.96 ± 0.35	17.24 ± 0.25	8.06 ± 0.10
Stearic acid	1.83 ± 0.07	2.08 ± 0.09	1.72 ± 0.09	1.53 ± 0.15	2.48 ± 0.10
Arachidic acid	0.83 ± 0.10	1.35 ± 0.07	0.36 ± 0.07	0.71 ± 0.09	nd
Oleic acid	26.64 ± 0.40	60.59 ± 0.27	17.06 ± 0.44	43.5 ± 1.11	82.39 ± 0.99
Linoleic acid	54.08 ± 0.31	22.38 ± 0.59	56.68 ± 0.61	35.53 ± 2.08	7.07 ± 0.06
Linolenic acid	6.57 ± 0.22	7.52 ± 0.07	0.55 ± 0.11	1.23 ± 0.12	nd
α-linolenic acid	nd	1.17 ± 0.05	nd	nd	nd
γ-linolenic acid	0.83 ± 0.21	nd	nd	nd	nd
SFA	11.86 ± 0.33	8.34 ± 0.27	25.71 ± 0.58	19.74 ± 0.5	10.53 ± 0.2
MUFA	26.64 ± 0.40	60.59 ± 0.27	17.06 ± 0.44	43.5 ± 1.11	82.39 ± 0.99
PUFA	61.48 ± 0.74	31.07 ± 0.71	57.23 ± 0.73	36.77 ± 2.2	7.07 ± 0.06
*Tocopherol isomer content (mg/100 mg)*					
α-tocopherol	5.56 ± 1.05	24.30 ± 4.02	2.69 ± 0.41	16.65 ± 0.95	22.46 ± 2.14
(β + γ)-tocopherol	66.20 ± 2.76	59.02 ± 0.97	82.86 ± 1.17	4.25 ± 1.00	2.79 ± 1.33
δ-tocopherol	23.33 ± 3.04	3.33 ± 1.04	50.66 ± 0.76	nd	nd
Total tocopherol	95.03 ± 1.25	86.65 ± 2.58	136.20 ± 2.03	20.90 ± 0.77	25.25 ± 3.23

Note: ‘nd’, not detected; SO, soybean oil; RO, rapeseed oil; CSO, cottonseed oil; RBO, rice bran oil; CO, camellia oil.

**Table 2:** Induction period (IP), protection factor (PF), and percentage antioxidant activity (%AA) of oil samples.

Indices Oil Samples	IP			PF			%AA		
	*Blank*	*RE*	*BHA + BHT*	*Blank*	*RE*	*BHA + BHT*	*Blank*	*RE*	*BHA + BHT*
SO	2.20 ± 0.22 ^c^	3.40 ± 0.18 ^a^	2.90 ± 0.12 ^b^	1	1.55	1.32	0	4.83	1
RO	2.32 ± 0.23 ^c^	4.15 ± 0.16 ^a^	3.05 ± 0.24 ^b^	1	1.79	1.31	0	5.71	1
CSO	1.88 ± 0.20 ^c^	3.35 ± 0.15 ^a^	2.84 ± 0.13 ^b^	1	1.79	1.51	0	3.49	1
RBO	3.83 ± 0.07 ^c^	6.22 ± 0.21 ^a^	4.79 ± 0.18 ^b^	1	1.62	1.25	0	6.50	1
CO	1.54 ± 0.28 ^c^	3.39 ± 0.26 ^a^	2.69 ± 0.20 ^b^	1	2.20	1.75	0	2.94	1

Note: Mean values in the same row followed by the same superscript letter are not significantly different (*p* > 0.05). SO, soybean oil; RO, rapeseed oil; CSO, cottonseed oil; RBO, rice bran oil; CO, camellia oil.

**Table 3:** Initial oxidation temperature (IOT) (°C) of oil samples under the five heating rates.

Oil Samples	Treatment	Heating Rate (°C/min)				
		3	6	9	12	15
SO	*Blank*	159.1 ± 0.3 ^c^	168.2 ± 0.6 ^c^	175.3 ± 0.2 ^c^	179.7 ± 0.5 ^c^	181.0 ± 1.1 ^c^
	*RE*	170.3 ± 0.4 ^a^	179.1 ± 0.3 ^b^	184.9 ±0.1 ^b^	191.3 ± 0.9 ^b^	197.1 ± 0.3 ^b^
	*BHA + BHT*	168.4 ± 0.7 ^b^	183.5 ± 0.8 ^a^	188.0 ± 1.2 ^a^	192.7 ± 0.2 ^a^	201.4 ± 1.3 ^a^
RO	*Blank*	163.8 ± 0.9 ^b^	179.4 ± 1.1 ^b^	185.7 ±0.4 ^b^	191.4 ± 0.8 ^b^	193.9 ± 0.2 ^b^
	*RE*	179.5 ± 0.6 ^a^	189.0 ± 0.7 ^a^	196.5 ± 0.6 ^a^	202.8 ± 1.1 ^a^	204.9 ± 0.3 ^a^
	*BHA + BHT*	159.4 ± 0.3 ^c^	169.5 ± 0.1 ^c^	178.8 ± 0.4 ^c^	179.9 ± 0.4 ^c^	187.3 ± 0.2 ^c^
CSO	*Blank*	145.0 ± 0.5 ^b^	159.1 ± 0.9 ^b^	165.6 ±1.4 ^b^	175.9 ± 0.2 ^b^	181.7 ± 0.8 ^b^
	*RE*	159.1 ± 0.2 ^a^	170.5 ± 0.4 ^a^	178.8 ± 0.8 ^a^	183.3 ± 0.6 ^a^	184.3 ± 1.1 ^a^
	*BHA + BHT*	145.4 ± 0.6 ^b^	154.6 ± 0.6 ^c^	163.3 ± 0.3 ^c^	170.6 ± 0.2 ^c^	177.0 ± 0.7 ^c^
RBO	*Blank*	171.9 ± 1.2 ^b^	183.7 ± 0.5 ^b^	192.5 ±0.7 ^b^	198.6 ± 0.4 ^a^	201.1 ± 0.3 ^ab^
	*RE*	177.1 ± 0.1 ^a^	187.9 ± 0.3 ^a^	194.8 ± 0.5 ^a^	197.8 ± 1.3 ^ab^	202.3 ± 1.3 ^a^
	*BHA + BHT*	171.1 ± 0.3 ^b^	183.7 ± 1.2 ^b^	187.3 ± 0.2 ^c^	196.4 ± 0.5 ^b^	199.0 ± 1.1 ^b^
CO	*Blank*	153.6 ± 0.8 ^b^	166.9 ± 0.4 ^c^	178.8 ±0.3 ^b^	185.9 ± 0.9 ^b^	193.0 ± 0.5 ^a^
	*RE*	159.2 ± 0.6 ^a^	177.7 ± 1.2 ^a^	183.0 ± 0.7 ^a^	188.1 ± 0.6 ^a^	190.4 ± 0.2 ^b^
	*BHA + BHT*	154.7 ± 0.2 ^b^	170.0 ± 0.9 ^b^	177.3 ± 0.1 ^c^	183.7 ± 0.3 ^c^	184.7 ± 0.4 ^c^

Note: Mean values in the same row followed by the same superscript letter are not significantly different (*p* > 0.05). SO, soybean oil; RO, rapeseed oil; CSO, cottonseed oil; RBO, rice bran oil; CO, camellia oil.

**Table 4:** Kinetic parameters (k at 180 °C) calculated from the initial oxidation temperature (IOT) under different heating rates.

Oil Samples	Treatment	*Ea* (kJ/mol)	A	k	Linear Regression Equation	R^2^
SO	*Blank*	84.81	2.12 × 10^9^	0.90	y = −4659x + 11.02	0.977
	*RE*	108.23	2.69 × 10^12^	0.35	y = −5945x + 14.23	0.991
	*BHA + BHT*	99.36	1.14 × 10^11^	0.40	y = −5458x + 12.82	0.980
RO	*Blank*	84.63	2.50 × 10^9^	0.44	y = −4649x + 11.09	0.987
	*RE*	103.82	2.03 × 10^11^	0.22	y = −5703x + 13.09	0.993
	*BHA + BHT*	91.84	2.64 × 10^10^	0.68	y = −5045x + 12.15	0.979
CSO	*Blank*	65.88	3.69 × 10^8^	0.94	y = −3619x + 9.15	0.988
	*RE*	93.84	4.49 × 10^10^	0.68	y = −5155x + 12.39	0.986
	*BHA + BHT*	74.58	4.70 × 10^8^	1.19	y = −4097x + 10.31	0.979
RBO	*Blank*	88.69	5.09 × 10^9^	0.30	y = −4872x + 11.42	0.995
	*RE*	109.08	9.49 × 10^11^	0.25	y = −5992x + 13.78	0.996
	*BHA + BHT*	93.86	2.30 × 10^10^	0.35	y = −5161x + 12.10	0.982
CO	*Blank*	63.64	1.32 × 10^8^	0.61	y = −3496x + 8.69	0.994
	*RE*	79.24	7.01 × 10^8^	0.51	y = −4353x + 10.51	0.965
	*BHA + BHT*	79.23	9.25 × 10^8^	0.68	y = −4352x + 10.63	0.984

Note: *Ea*, activation energy; A, pre-exponential factor; k, reaction rate constant. SO, soybean oil; RO, rapeseed oil; CSO, cottonseed oil; RBO, rice bran oil; CO, camellia oil.

## Data Availability

The data are available from the corresponding author.

## Supplementary Materials

The following supporting information can be downloaded at https://www.mdpi.com/article/10.3390/foods12112177/s1: Figure S1: Fitting curves calculated by the O–F–W equation and Arrhenius equation from IOTs under different heating rates of each sample.

## Abbreviations

AA, antioxidant activity; ABTS, 2,2′-azino-bis(3-ethylbenzothiazoline-6-sulfonic acid); ANOVA, analysis of variance; BHA, butylated hydroxyanisole; BHT, butylated hydroxytoluene; CO, camellia oil; CSO, cottonseed oil; DPPH, 1,1-diphenyl-2-picrylhydrazyl; DSC, differential scanning calorimetry; GRAS, generally recognized as safe; IOT, initial oxidation temperature; IP, induction period; MUFA, monounsaturated fatty acid; OFW, Ozawa–Flynn–Wall; PF, protection factor; PUFA, polyunsaturated fatty acid; RBO, rice bran oil; RE, rosemary (*Rosmarinus officinalis* L.) extract; RO, rapeseed oil; SFA, saturated fatty acid; SO, soybean oil; TPC, total phenolic content; *Ea*, activation energy; A, pre-exponential factor; k, reaction rate constant.
