# Supplementary material for: Protection Function and Mechanism of Rosemary (Rosmarinus officinalis L.) Extract on the Thermal Oxidative Stability of Vegetable Oils
Source: Foods. 2023 May 28;12(11):2177. doi: 10.3390/foods12112177 (PMC10252516; doi:10.3390/foods12112177)
Supplement: Supplementary file 1 [file foods-12-02177-s001.zip › foods-2395446-supplementary.pdf]

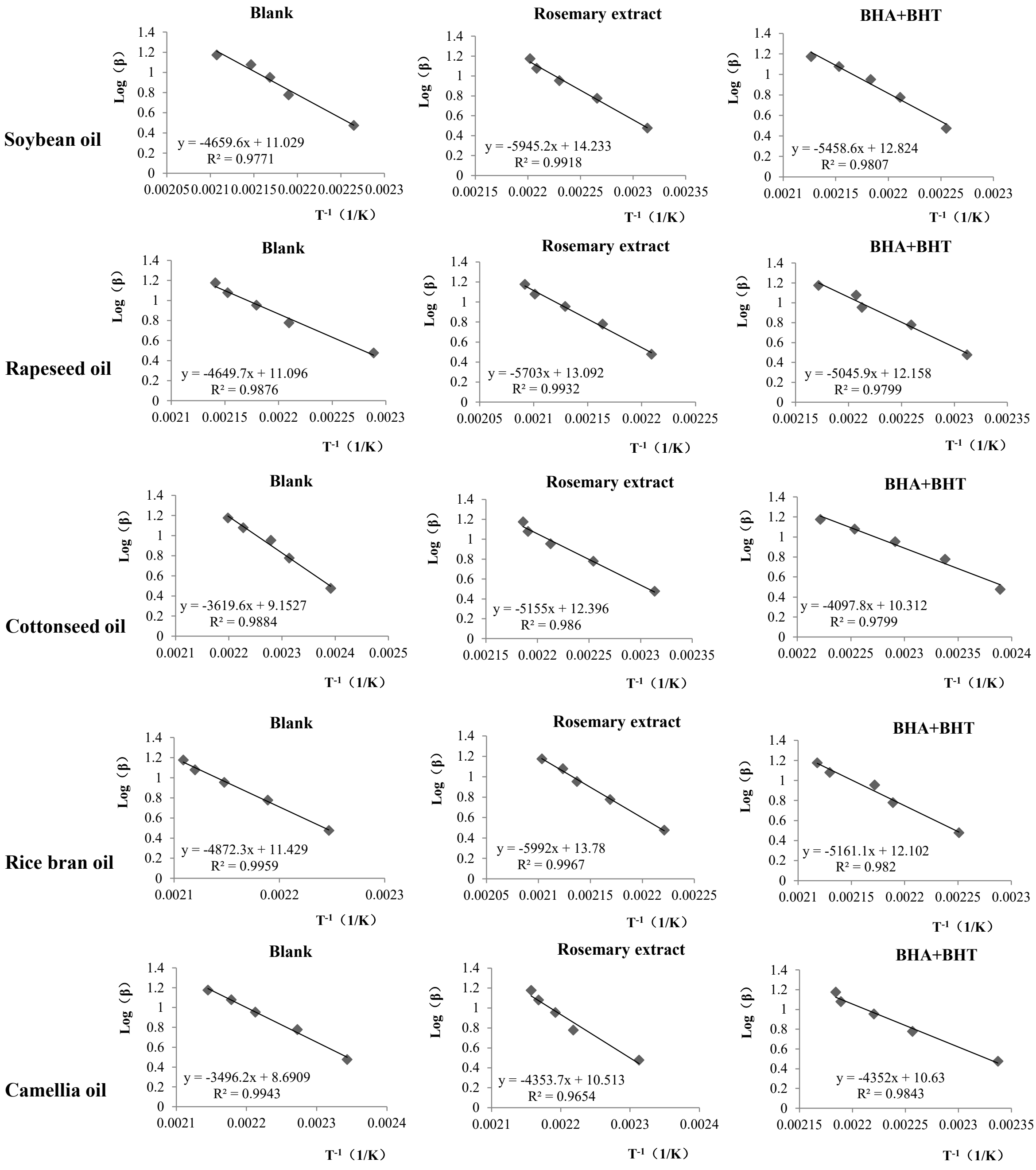


**Figure S1.** Scatter fitting graph calculated by O-F-W equation and Arrhenius equation from IOTs under different heating rates of each sample.
